# Cost of the quantitative adequacy of nursing staff in the ICU

**DOI:** 10.1186/cc12624

**Published:** 2013-06-19

**Authors:** CP Guimarães, PC Garcia, FMT Fugulin

**Affiliations:** 1 Escola de Enfermagem da Universidade de São Paulo, SP, Brazil; 2 Hospital Universitário da Universidade de São Paulo, SP, Brazil

## Introduction

The high cost to maintain a complex structure such as the ICU has justified its strict control. Nevertheless, budgetary limitation and expenses abatement directly affect the outcome of a fully adequate nursing staff due to its high-percentage representation in human resources. Therefore, the lack of proper personnel interferes on many aspects of daily activities such as organization and safety of patients and staff, affecting also the assistance provided and the institutional goals. For that reason, study on costs and management of nursing personnel is paramount, as it can evidence the effects of an impaired scenario and the relation between cost and efficiency in a healthcare environment. The objectives were: to verify the average staff time required by a patient for a proper assistance or treatment; to calculate the actual average time and cost spent by the crew; and to estimate the average daily time and cost for a proper adult ICU's activity.

## Methods

The research was based on a quantitative and descriptive data. The study took place in the Hospital Universitário da Universidade de São Paulo's adult ICU block, from 1 January 2008 to 31 December 2009. Data concerning the average time of assistance given to the patients and requested by them were collected from the ICU's management instruments. Data concerning personnel fees per hour were based on the ICU nursing staff's wage bill, provided by the Finance Department.

## Results

The daily average time of required assistance is 16 hours. However, the actual daily average time of provided assistance is 14 hours, which poses a great disparity statistically. In 24 hours the average cost of given assistance per patient was R$715.79. On the other hand, adequate assistance would require R$805.66. The average cost per month to amend the actual scenario would be R$40,490.00, which corresponds to an increase of 17.16% over the existing outline's budget.

## Conclusion

The literature review and the data suggest that although the adequacy of the nursing staff entails higher costs, it may contribute to improve the quality of care, reducing costs arising from possible negative outcomes in patients.
